# Chronic Changes on Kidney Histology by a Multiclass Artificial Intelligence Model

**DOI:** 10.1016/j.ekir.2025.05.035

**Published:** 2025-05-29

**Authors:** Aleksandar Denic, Muhammad S. Asghar, Lucas Stetzik, Austin Reynolds, Jaidip M. Jagtap, Mahesh Kumar, Aidan F. Mullan, Andrew R. Janowczyk, Mariam P. Alexander, Maxwell L. Smith, Fadi E. Salem, Laura Barisoni, Andrew D. Rule

**Affiliations:** 1 Division of Nephrology and Hypertension, Mayo Clinic, Rochester, Minnesota, USA; 2 Aiforia Inc, Cambridge Innovation Center, Cambridge, Massachusetts, USA; 3 Department of Radiology, Mayo Clinic, Rochester, Minnesota, USA; 4 Division of Biomedical Statistics and Informatics, Mayo Clinic, Rochester, Minnesota, USA; 5 Department of Biomedical Engineering, Emory University and Georgia Institute of Technology, Atlanta, Georgia, USA; 6 Division of Precision Oncology, Department of Oncology, Geneva University Hospitals, Geneva, Switzerland; 7 Department of Diagnostics, Division of Clinical Pathology, Geneva University Hospitals, Geneva, Switzerland; 8 Department of Laboratory Medicine and Pathology, Mayo Clinic, Rochester, Minnesota, USA; 9 Department of Laboratory Medicine and Pathology, Mayo Clinic, Scottsdale, Arizona, USA; 10 Department of Laboratory Medicine and Pathology, Mayo Clinic, Jacksonville, Florida, USA; 11 Department of Pathology, Division of AI and Computational Pathology, Duke University, Durham, North Carolina, USA; 12 Department of Medicine, Division of Nephrology, Mayo Clinic, Rochester, Minnesota, USA; 13 Division of Epidemiology, Mayo Clinic, Rochester, Minnesota, USA

**Keywords:** artificial intelligence, chronic kidney disease, digital pathology, kidney histology, nephron size, nephrosclerosis

## Abstract

**Introduction:**

Chronic changes in kidney histology are often approximated by using human vision but with limited accuracy.

**Methods:**

An interactive annotation tool trained an artificial intelligence (AI) model for segmenting structures on whole slide images (WSIs) of kidney tissue. A total of 20,509 annotations trained the AI model with 20 classes of structures, including separate detection of cortex from medulla. We compared the AI model detections with human-based annotations in an independent validation set. The AI model was then applied to 1426 donors and 1699 patients with renal tumor to calculate chronic changes as defined by measures of nephron size (glomerular volume, cortex volume per glomerulus, and mean tubular areas) and nephrosclerosis (globally sclerotic glomeruli, increased interstitium, increased tubular atrophy (TA), arteriolar hyalinosis (AH), and artery luminal stenosis from intimal thickening). We then assessed whether chronic kidney disease (CKD) outcomes were associated with these chronic changes.

**Results:**

During the AI model validation step, the agreement between the AI detections and human annotations was similar to the agreement between human pairs, except that the AI model showed less agreement with AH. Chronic changes calculated solely from AI-based detections associated with low glomerular filtration rate (GFR) during follow-up after kidney donation and with kidney failure after a radical nephrectomy for tumor. A chronicity score based on AI detections was calculated from cortex per glomerulus, percent glomerulosclerosis, TA foci density, and mean area of AH lesions and showed good prognostic discrimination for kidney failure (cross-validation C-statistic = 0.819).

**Conclusion:**

A multiclass AI model can help automate quantification of chronic changes on WSIs of kidney histology.

## Graphical abstract

**Figure:**
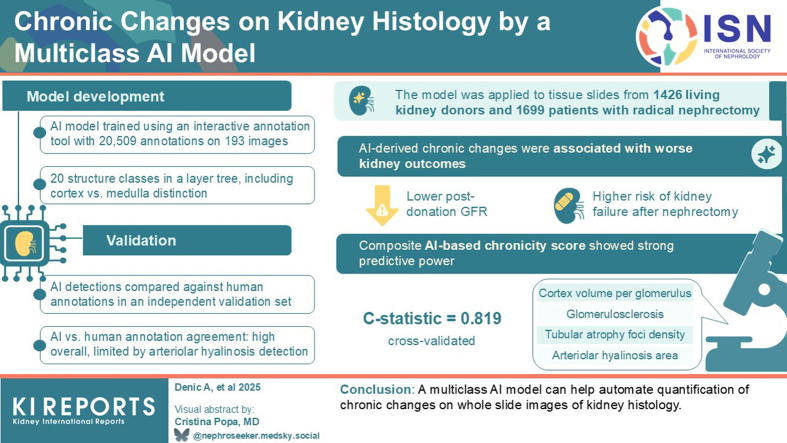

See Commentary on Page 2535

Larger nephron size (higher glomerular volume, cortex volume per glomerulus, and tubular cross-sectional area) and nephrosclerosis (global glomerulosclerosis, interstitial fibrosis, TA, arteriosclerosis, and AH) are indicators of chronic tissue damage (chronic changes) that are linked to proteinuria and low GFR and are associated with CKD progression.^1^, ^2^, ^3^, ^4^, ^5^, ^6^, ^7^, ^8^ Kidney histological assessment of these chronic changes has been achieved via human vision using a semiquantitative scale or via morphometry using human drawn annotations of individual structures on WSI. Although visual assessment is practical in clinical practice, it has suboptimal interobserver and intraobserver reproducibility.^9^ In contrast, manual morphometry is more reproducible and precise^10^; however, it is time consuming and cannot be applied in the clinical setting. We recently demonstrated that manual morphometry with annotations to quantify chronic changes on routine “for cause” native kidney biopsy tissue on a single Periodic acid Schiff (PAS)-stained section is comparable with visual estimates in predicting progressive CKD.^2^ Manual morphometry also quantifies chronic changes highly predictive of progressive CKD that are generally not reported (e.g., count density of interstitial fibrosis and TA foci) or simplified when reported (e.g., AH) on clinical pathology reports.^11^

The application of AI to images of kidney tissue has the potential to improve our ability to reproducibly and rapidly quantify structural pathology that is clinically relevant, resulting in less burdensome work for pathologists and improved health care. In recent years, AI has been applied to kidney tissue images to segment structures in a variety of patient populations.^12^, ^13^, ^14^, ^15^, ^16^, ^17^, ^18^, ^19^ These previous studies have shown good agreement (intersection) statistics between human annotations and AI detections of the same structure. However, existing AI models have not been comprehensive enough to sufficiently detect the structures needed to quantify chronic changes on kidney tissue. Therefore, we trained and tested a 20-class AI model to segment the major structures and their subclasses to morphometrically quantify chronic changes on images of kidney tissue. We then assessed whether the chronic changes calculated from this AI model were predictive of CKD outcomes in living kidney donors and in patients with a renal tumor.

## Methods

### Study Samples

This study was approved by the Institutional Review Board of Mayo Clinic and conducted in accordance with the ethical standards outlined in the Declaration of Helsinki. The Aging Kidney Anatomy study included images from living kidney donors between 2000 and 2015 (Mayo Clinic Minnesota and Arizona) and from patients with a renal tumor who underwent radical nephrectomy between 2000 and 2021 (Mayo Clinic, Minnesota). Selection of living kidney donors was based on their health, and although criteria for donation varied by era and site, these criteria required 24-h urine albumin < 30 mg/24 h and a measured GFR (mGFR) normal for age.^20^ Further, older donors with mild hypertension and moderate obesity (body mass index: 30–35 kg/m^2^ and rarely up to 40 kg/m^2^) were allowed to donate. Mild hypertension was defined as an office systolic blood pressure of 140 to 159 mm Hg, diastolic blood pressure of 90 to 99 mm Hg, or use of 1 antihypertensive medication (with or without a thiazide diuretic). Potential donors with more severe hypertension, diabetes, or cardiovascular disease were not allowed to donate. None of the patients with renal tumor in this study had metastatic lesions or positive lymph nodes at the time of surgery. We excluded patients with a diffuse specific kidney disease on histology, diffuse tubulointerstitial inflammation (throughout both cortex and medulla), diffuse interstitial fibrosis and TA (affecting > 90% of cortex), a large focal scar, or end-stage kidney histology (thin and completely scarred cortex).^3^ Patients who had evidence of mild to moderate diabetic nephropathy were not excluded.

### Clinical Characteristics

Baseline clinical characteristics (age, sex, body mass index, serum creatinine [corrected to standardized values if assayed prestandardization], hypertension, and diabetes) and diagnoses of both cohorts were based on review of medical records at the time of nephrectomy. Estimated GFR (eGFR) was measured using the serum creatinine–based CKD-Epidemiology Collaboration equation,^21^ and mGFR (only available in donors) was measured by using urinary iothalamate clearance.^22^ The 24-h urine protein was estimated from urine protein to osmolality ratios as previously described.^23^

### Kidney Biopsy and WSIs

**Figure 1:**
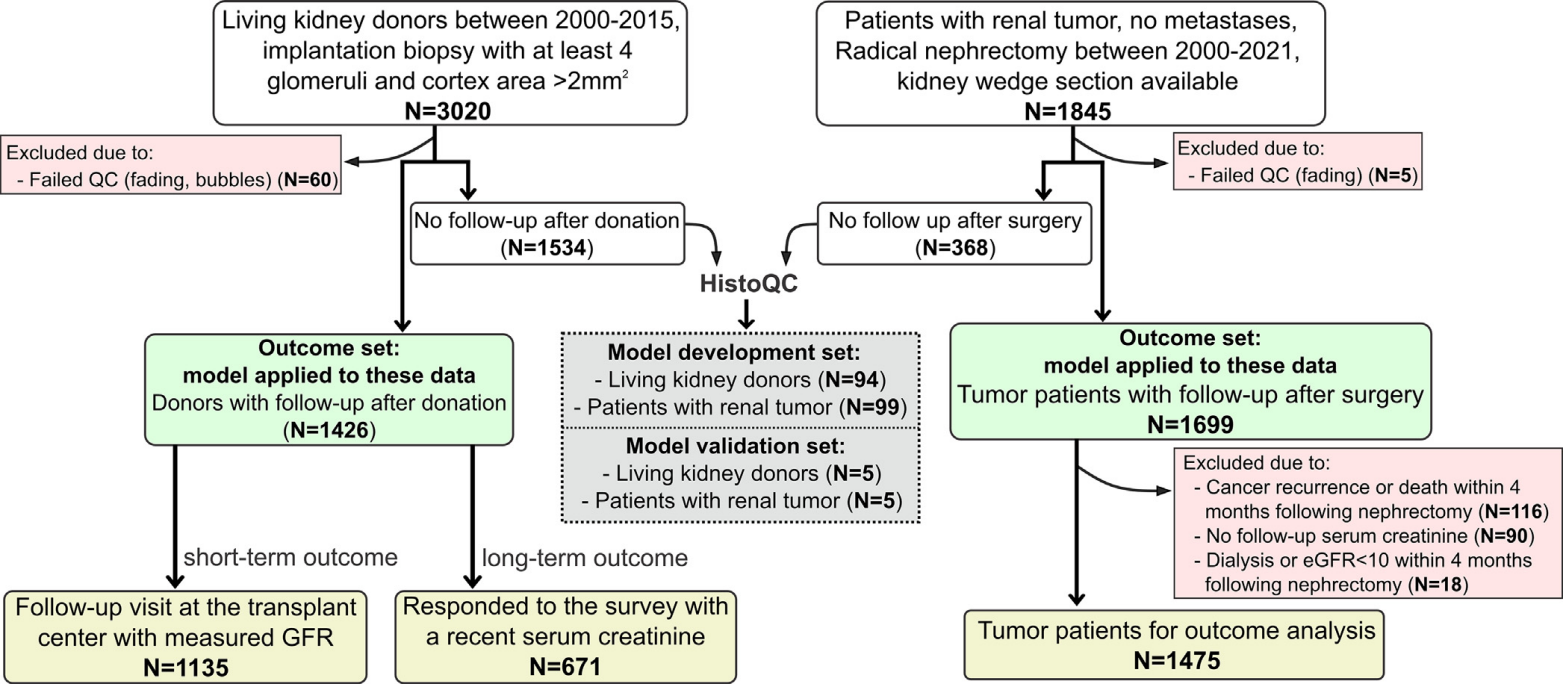
Flowchart shows the selection of model development and outcome sets for living kidney donors and patients with a renal tumor. eGFR, estimated glomerular filtration rate; QC, quality check

WSIs of kidney tissue from the Aging Kidney Anatomy study^1^^,^^3^^,^^6^ were used to develop the AI model (Figure 1). Kidney tissue was obtained from the kidney removed at the time of the nephrectomy in both cohorts. In living kidney donors, a needle core biopsy was obtained; whereas with patients with a renal tumor, a wedge section was obtained of the nontumor kidney parenchyma that was away from the tumor. A 2- to 3-μm thick section was stained with PAS and scanned into a WSI using an Aperio scanner. All images were at 20× magnification (∼0.5 micron per pixel). To ensure the model was trained across the spectrum of kidney images available (e.g., differences in staining protocols or scanner used), HistoQC^14^ was applied to images from kidney donors and patients with tumor to select 193 images that were representative of the variability of kidney biopsy images in both cohorts. A separate set of 10 patients (5 living kidney donors and 5 patients with tumor) were used for validation of the AI model. The final model was then applied to an independent set of patients not used for model development that included 1426 living kidney donors and 1699 patients with a renal tumor with follow-up for outcomes and with images that lacked severe fading or other artifacts on quality control review (Figure 1).

### AI Model

**Figure 2:**
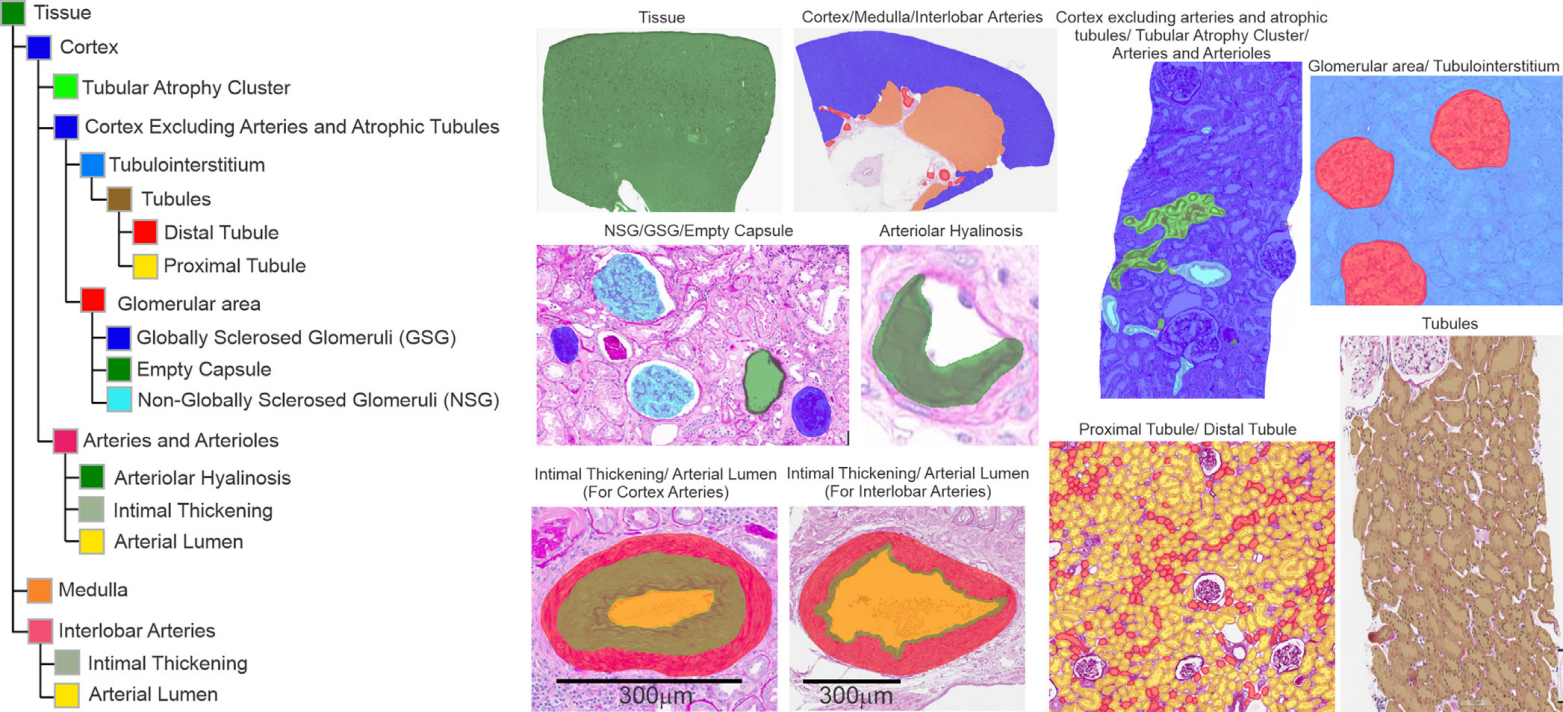
The multilayer 20 class AI model with examples of detections for each class. AI, artificial intelligence.

The AI model was developed using Aiforia Create, an interactive tool that iteratively trains a multi-class AI model from annotations without the requirement of coding.S1–S3 Details regarding the development, validation, and extraction of quantitative data of the AI model are included in the Supplementary Methods. Briefly, the model had 20 classes that covered the major glomerular, tubulointerstitial, and arterial structures and their chronic changes (Figure 2). A total of 20,509 annotations were used to train 20 classes of structures in the final model (Supplementary Figures S1–S3). Model validation was performed by 7 individuals (AD, SMA, LB, MLS, MPA, FES, and ADR) annotating representative examples of all 20 classes of structures across images from the separate set of 10 patients (Supplementary Methods). Intersection statistics between the AI model detections of structures and human annotations of structures were similar to that between human pairs, except for AH, which had stronger agreement between human pairs (Table 1). After validation, the AI model was applied to segment structures in the 1426 donors and 1699 patients with tumor with follow-up for CKD outcomes after excluding those with fading or other artifacts on quality control. Measurements were then extracted from the images of segmented structures without any manual or computer-aided editing and without exclusion of any images with AI detection errors. Extracted data also included the automated identification of segmented cortical structures (glomeruli, arteries, and TA clusters) adjacent to the cortex edge, but not the kidney capsule, and thus bisected by the biopsy needle.S4–S6

**Table 1:** Validation results with each class of the AI model against 7 human annotators compared with between-7 human annotators

Class	Comparison	False positive, %	False negative, %	Precision, %	Sensitivity, %	F1 score, %
Tissue	AI vs. human	0.0	0.0	99.6	99.8	99.7
	Human vs. human	0.0	0.0	99.8	99.8	99.8
Cortex	AI vs human	0.2	0.2	96.6	97.9	96.8
	Human vs. human	0.2	0.1	97.0	97.0	96.1
Tubular atrophy cluster	AI vs. human	1.5	0.5	75.8	94.9	84.6
	Human vs. human	1.1	1.0	84.8	84.8	85.6
Cortex excluding arteries and atrophic tubules	AI vs. human	0.0	0.1	99.7	98.1	98.9
	Human vs. human	0.1	0.1	99.1	99.1	99.1
Tubulointerstitium	AI vs. human	0.5	0.3	95.7	96.5	96.0
	Human vs. human	0.5	0.5	95.5	95.5	95.4
Tubules	AI vs. human	0.2	0.1	97.3	98.7	99.2
	Human vs. human	0.2	0.2	96.9	96.9	99.5
Distal tubule	AI vs. human	12.8	9.3	86.7	90.7	92.8
	Human vs. human	6.6	8.5	91.0	91.0	97.5
Proximal tubule	AI vs. human	12.3	3.0	74.1	97.0	95.5
	Human vs. human	2.3	4.7	95.8	95.8	99.5
Glomerular area	AI vs. human	0.7	0.1	91.6	98.8	96.3
	Human vs. human	0.5	0.5	94.3	94.3	96.7
Globally sclerosed glomeruli	AI vs. human	4.2	1.5	91.1	98.5	97.9
	Human vs. human	4.6	5.2	94.6	94.6	97.5
Empty capsule	AI vs. human	1.7	7.0	84.3	93.0	99.0
	Human vs. human	9.8	13.9	85.5	85.5	98.2
Nonglobally sclerosed glomeruli	AI vs. human	0.6	1.2	96.5	98.8	99.4
	Human vs. human	1.7	4.4	95.5	95.5	98.8
Arteries and arterioles	AI vs. human	0.8	0.1	81.3	97.6	90.5
	Human vs. human	0.5	0.4	88.8	88.8	92.0
Arteriolar hyalinosis	AI vs. human	4.9	0.4	50.4	94.8	64.1
	Human vs. human	1.5	1.1	80.0	80.0	79.8
Intimal thickening	AI vs. human	0.4	0.5	91.5	82.7	92.8
	Human vs. human	0.4	0.4	92.3	92.3	92.0
Arterial lumen	AI vs. human	0.2	0.6	87.4	81.6	95.9
	Human vs. human	0.2	0.2	93.7	93.7	96.8
Medulla	AI vs. human	0.2	0.2	87.0	93.9	97.6
	Human vs. human	0.1	0.1	94.8	94.8	97.4
Interlobar arteries	AI vs. human	0.2	0.2	96.1	86.9	96.7
	Human vs. human	0.2	0.2	87.2	87.2	97.6
Intimal thickening	AI vs. human	3.6	1.0	82.6	95.1	87.6
	Human vs. human	2.5	2.4	87.8	87.8	86.3
Artery lumen	AI vs. human	0.2	0.3	98.6	98.5	98.5
	Human vs. human	0.3	0.3	98.3	98.3	98.2

AI, artificial intelligence.

### Chronic Changes Derived From AI Detections

Using the AI model detections for each structure, we calculated measures of nephron size and nephrosclerosis in the donor (*n* = 1426) and patient with tumor (*n* = 1699) cohorts with follow-up.^1^^,^^4^^,^^6^^,^ S7 Details on how AI detections were used to calculate chronic changes are presented in Supplementary Table S1. Briefly, the AI detected all TA clusters and all interstitium (fibrosis or not). Because the AI model could not distinguish with certainty whether interstitium was fibrotic or not, we reported the fraction of the tubulointerstitium that was interstitium and TA (%ITA) rather than percent interstitial fibrosis and TA. The AI detected the presence and area of AH within all arterioles; however, if 2 AH lesions were < 500 μm apart (about the width of 2 adjacent glomerular profiles), only 1 was counted. Finally, arteriosclerosis was based on the percent lumen stenosis from intimal thickening averaged across all arteries with an area larger than 7850 μm^2^S8 as individually detected by the AI model. The AI model detected intima only when thickened.

### Outcomes

Per standardized clinical protocol, the living kidney donors returned to their donation center for a follow-up visit a median of 4.4 months after donation, at which time mGFR was performed by using urinary iothalamate clearance and CKD was identified by an mGFR < 45 ml/min per 1.73 m^2^.^7^ The kidney donors were also surveyed a median of 10.5 years postdonation to obtain their serum creatinine, which was then used to identify CKD by an eGFR < 45ml/min per 1.73 m^2^.^4^ Patients with a renal tumor had a follow-up visit every 3 to 6 months during the first year postnephrectomy, and then every 6 to 12 months. The final follow-up visit in this study was on September 1, 2023. Per a standardized clinical protocol, serum creatinine level was included with these follow-up visits. If patients could not return to Mayo Clinic, they were surveyed by phone to determine serum creatinine levels, dialysis status, or kidney transplantation status. We used serum creatinine levels closest to, but before 4 months following the nephrectomy to define the postnephrectomy baseline eGFR.^6^ We defined an outcome of progressive CKD by onset of dialysis, kidney transplantation, a 40% decline in eGFR from the postnephrectomy baseline sustained for at least 3 months, or by an eGFR < 10 ml/min per 1.73 m^2^ and at least 5 ml/min per 1.73 m^2^ below postnephrectomy baseline sustained for at least 3 months.^24^ We defined kidney failure as the first date of dialysis, kidney transplantation, or onset of eGFR < 10 ml/min per 1.73 m^2^.

### Statistical Analysis

We have previously reported how chronic changes based on simplified manual annotations associated with these outcomes in both the donors^4^^,^^7^^,^^25^^,^^26^ and the patients with a renal tumor.^3^^,^^6^^,^^24^^,^^26^ Therefore, we performed a similar analysis, but using chronic changes based on AI model detections instead of manual annotations. Some of the chronic changes detected with the AI model were novel to this analysis. Specifically, we report clinical associations with AH, interstitium, TA clusters, and proximal and distal tubular area not previously reported in these data. We used Spearman’s correlations to compare the measures of nephron size and nephrosclerosis between AI biopsy measures and clinical characteristics and kidney function in both cohorts. Nephron size and nephrosclerosis measures were standardized (per SD) for assessing their association with CKD outcomes. We used Cox proportional hazard models for radical nephrectomy patients and logistic regression models for living kidney donors to assess the unadjusted and adjusted risk of CKD outcomes with measures of nephron size and nephrosclerosis. In donors, we used age, sex, baseline eGFR, and follow-up time; whereas in patients with tumor we used age, sex, body mass index, hypertension, diabetes, baseline eGFR, and proteinuria as covariates in adjusted analyses. In Cox models, censoring was performed at the last available serum creatinine, cancer recurrence, the last follow-up visit, or patient’s death. We created two 4-variable chronicity scores for the patients with tumor, one that was based on our previously developed score in patients with native kidney disease using manual annotations^2^ and the second that used LASSO to select the variables that predicted kidney failure from a multivariable model. We calculated the C statistic without and with 10-fold cross-validation for each chronicity score using Cox proportional hazard models. All statistical analyses were performed using BlueSky Statistics software version 10.3.4 (BlueSky Statistics LLC, Chicago, IL) and R (RStudio) version 4.3.2.

## Results

### Clinical Characteristics and Chronic Changes on Histology

**Figure 3:**
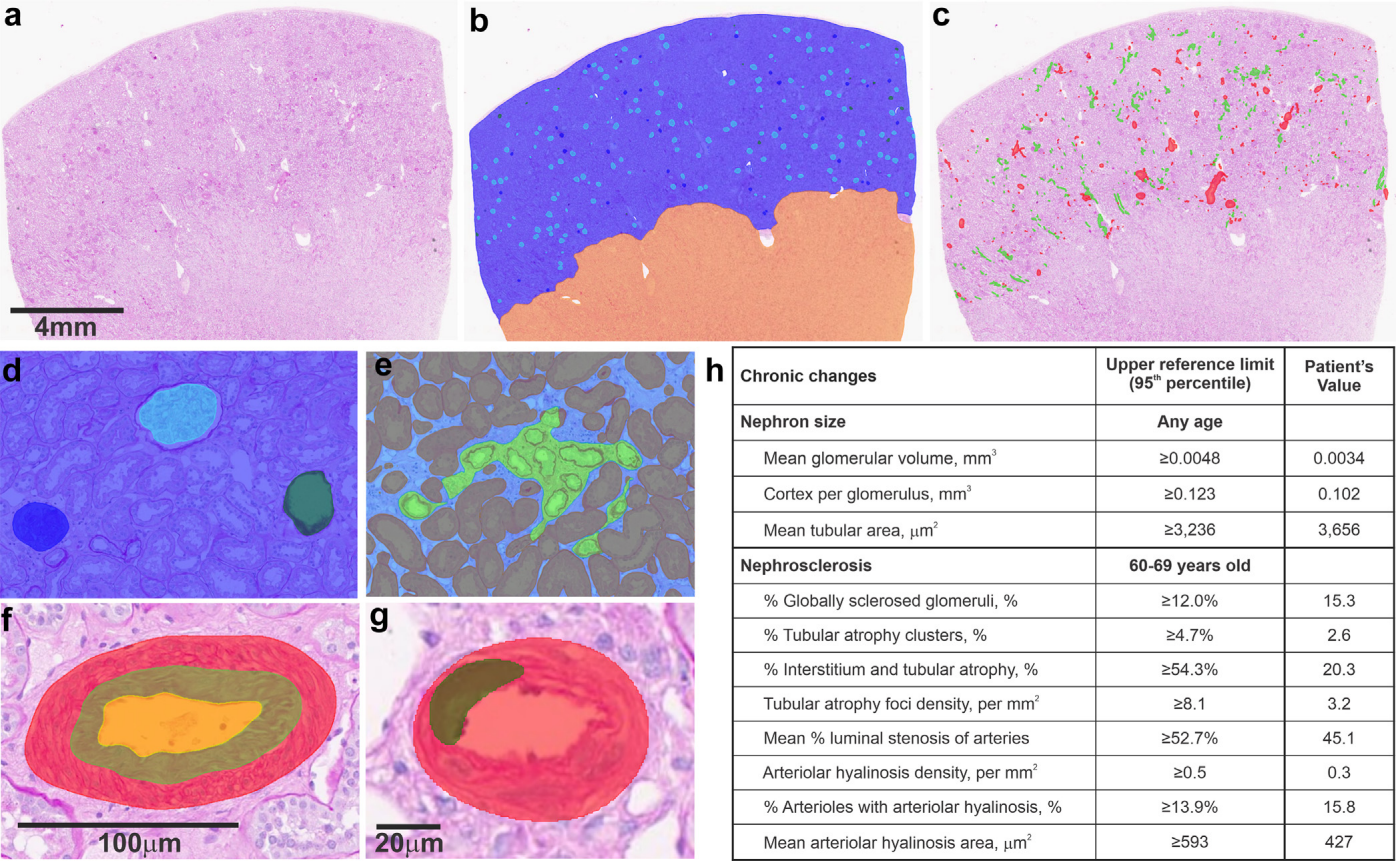
An example of image analysis and a pathology report of chronic changes in a 71-year-old patient with a renal tumor. (a) The original PAS-stained section was (b) overlaid with AI-detected cortex, medulla, and 3 glomerular classes, and tubular atrophy clusters (TA) (light green) and (c) all arteries and arterioles (red). (d) An example of a nonsclerosed glomerulus (cyan), globally sclerosed glomerulus (dark blue) and empty capsule (dark green). (e) An enlarged example of TA (light green), interstitium (light blue), and tubule area (brown). (f) An example of an artery (red) with intimal thickening (green) and lumen (yellow). (g) An example of an arteriole (red) with arteriolar hyalinosis (AH) lesion (dark green). (h) A pathology report of AI-calculated measures of nephron size and nephrosclerosis that includes upper reference limits (based on the living kidney donors) and patient’s value. AI, artificial intelligence; PAS, Periodic acid Schiff.

**Figure 4:**
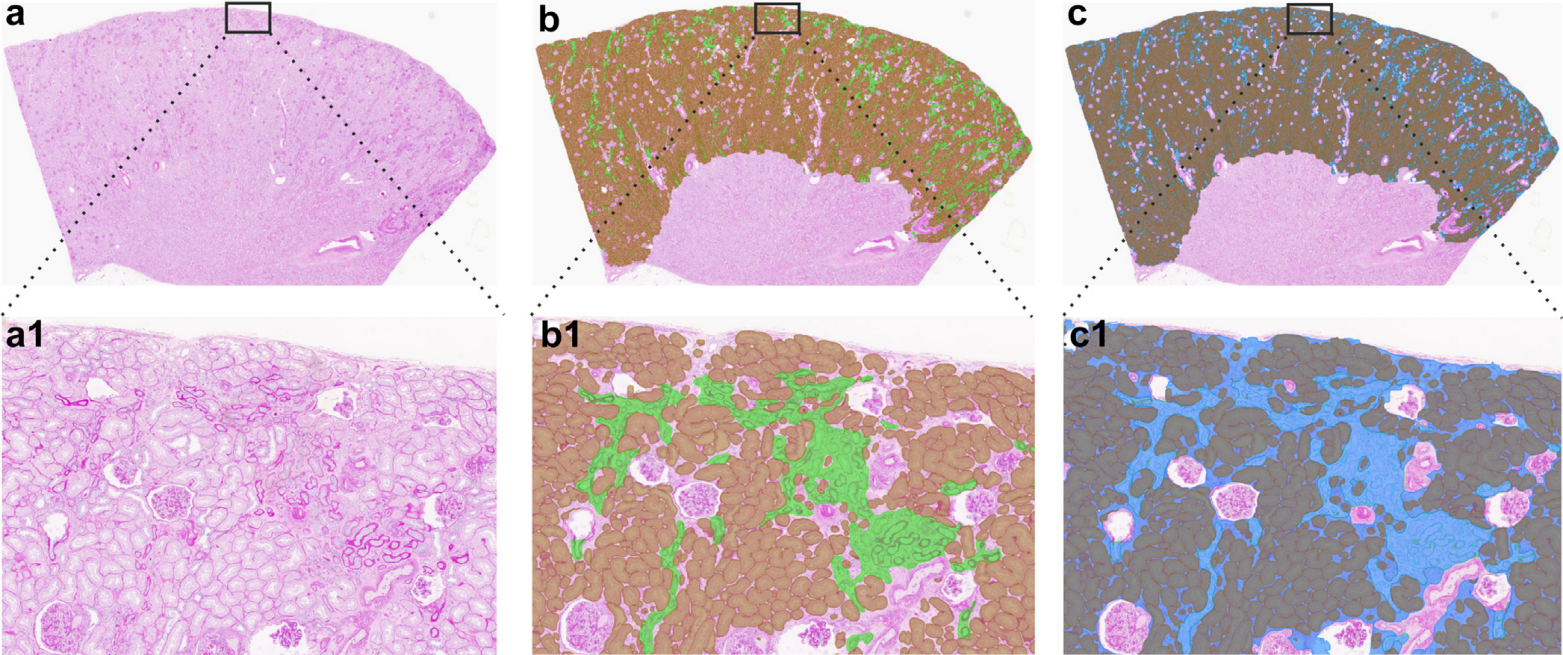
An example of the whole and magnified views of the same PAS-stained wedge section with AI detections used to calculate measures of tubular atrophy. (a) A clean image shown without AI detections. (b) AI-derived detections of tubular atrophy clusters (TA) (green) and all tubules (brown) that were used to calculate %TA per tubular area. (c) AI-derived detections of interstitium and TA cluster (ITA) (blue), and all tubules (brown) that were used to calculate %ITA per tubulointerstitial area. AI, artificial intelligence; PAS, Periodic acid Schiff.

In Table 2, we show the demographic and clinical characteristics of both cohorts at baseline (time of nephrectomy). Compared with kidney donors, patients with tumor were a mean of 18 years older, proportionally more male, more obese, more likely to be hypertensive or diabetic, and had a lower GFR and higher proteinuria. Figure 3 is an example of AI model detections in a patient with a renal tumor and the corresponding report of nephron size and nephrosclerosis measures along with reference ranges (based on living kidney donors). An example comparing how AI detections were used to calculate %TA per tubular area and %ITA per tubulointerstitial area on the same image is shown in Figure 4. The AI-detection derived chronic changes in 1426 living kidney donors and 1699 patients with tumor are shown in Table 3. Patients with tumor had similar glomerular volume, larger mean proximal and distal tubular areas, higher percentage of globally sclerosed glomeruli and TA, higher TA foci density, higher AH density, higher percentage of arterioles with AH, larger mean AH area, and higher percentage of luminal stenosis than living kidney donors.

**Table 2:** Clinical characteristics of living kidney donors and patients with a renal tumor

Clinical characteristic	Kidney donors (*n* = 1426)	Patients with tumor (*n* = 1699)	*P*-value
	mean (SD), median (IQR), or *n* (%)		
Demographic			
Age, yr	45.6 (11.8)	63.8 (12.1)	< 0.001
Men, %	554 (38.8%)	1120 (65.9%)	< 0.001
Race			0.17
White or unknown	1354 (94.9%)	1627 (95.8%)	
Black	25 (1.8%)	23 (1.4%)	
American Indian / Alaskan native	13 (0.9%)	20 (1.1%)	
Asian	16 (1.1%)	5 (0.3%)	
Other	18 (1.3%)	24 (1.4%)	
CKD risk factors			
Diabetes mellitus, %	0 (0%)	256 (15.1%)	< 0.001
Hypertension, %	233 (16.3%)	1142 (67.2%)	< 0.001
Body mass index, kg/m^2^	27.7 (4.8)	30.8 (6.8)	< 0.001
Presurgery kidney function			
amGFR, ml/min per 1.73 m^2^	102.7 (18.8)	N/A	
eGFR, ml/min per 1.73 m^2^	98.3 (15.9)	71.4 (19.8)	< 0.001
b24-h urine protein, mg	64 (46–91)	171 (96–358)	< 0.001
Postsurgery baseline kidney function			
ceGFR, ml/min per 1.73 m^2^	N/A	50.9 (14.4)	

CKD, chronic kidney disease; eGFR, estimated glomerular filtration rate; IQR, interquartile range; mGFR, measured glomerular filtration rate; N/A, not applicable.

a Data available in 1404 donors.

b Data available in 1421 donors and 1419 patients with tumor.

c Data available in 1571 patients with tumor.

**Table 3:** A summary of histological measures in living kidney donors (*n* = 1426) and patients with a renal tumor (*n* = 1699) by AI model

Histology measures	Donors Mean (SD)	Patients with tumor Mean (SD)
Select structural measures		
Cortex area, mm^2^	6.7 (2.4)	135.1 (55.9)
NSG number	16.5 (8.0)	334.4 (152.4)
Empty capsule number	2.9 (2.4)	22.2 (18.5)
GSG number	0.6 (1.1)	39.2 (42.8)
Number of TA foci	5.6 (6.6)	342.7 (340.1)
AH number	0.7 (1.2)	53.0 (56.6)
Measures of nephron size (calculated from structural measures)		
Glomerular volume, mm^3^	0.0028 (0.0010)	0.0027 (0.0008)
Cortex per glomerulus, per mm^3^	0.068 (0.034)	0.070 (0.029)
Mean proximal tubular area, μm^2^	2 768 (703)	4 205 (888)
Mean distal tubular area, μm^2^	1 523 (360)	2 598 (523)
Measures of nephrosclerosis (calculated from structural measures)		
%GSG, %	3.4 (5.7)	10.4 (9.4)
%TA or %ITA		
TA area per tubular area, %	0.7 (1.3)	4.1 (7.6)
Interstitium and TA area per tubulointerstitial area, %	36.5 (7.4)	24.1 (9.9)
TA foci density, per mm^2^		
TA foci density per tubular area	1.6 (2.0)	4.0 (3.9)
Vascular changes		
AH density per cortex area, per mm^2^	0.10 (0.18)	0.38 (0.32)
Arterioles with AH, %	2.5 (4.5)	12.4 (9.5)
Mean AH area, μm^2^	138 (236)	405 (146)
aLuminal stenosis of arteries, %	16.3 (18.2)	27.7 (12.9)

AH, arteriolar hyalinosis; AI, artificial intelligence; GSG, globally sclerosed glomeruli; IFTA, interstitial fibrosis and tubular atrophy; ITA, interstitium and tubular atrophy; NSG, nonsclerosed glomeruli; TA, tubular atrophy.

a Missing in 102 kidney donor biopsies by AI.

### Correlation of AI-Derived Chronic Changes With Clinical Characteristics

In donors and patients with a tumor, measures of larger nephron size were associated with male, higher body mass index, hypertension, and diabetes (Supplementary Table S2). Larger cortex per glomerulus and distal tubular area were associated with lower eGFR in patients with a tumor and larger proximal tubular area associated with higher eGFR in donors. Larger nephrons were associated with proteinuria in patients with a tumor but not donors. Measures of nephrosclerosis were associated with older age, hypertension, diabetes, lower eGFR in donors and patients with a tumor. Measures of nephrosclerosis were associated with male and proteinuria, but more consistently in patients with a tumor than donors.

### Association of AI-Derived Chronic Changes With CKD Outcomes

There were 42 of 1135 kidney donors with a mGFR < 45 ml/min per 1.73 m^2^ at a median of 4.4 months postdonation. There were 32 of 671 kidney donors with eGFR < 45 ml/min per 1.73 m^2^ at a median of 10.5 years postdonation. In general, nephrosclerosis measures were associated with the higher risk of both outcomes; however, several measures of nephron size were only associated consistently with long-term eGFR < 45 ml/min per 1.73 m^2^ (Table 4). Many of these associations were attenuated adjusting for clinical characteristics, but larger glomeruli and cortex per glomerulus and larger mean AH area still predicted an eGFR < 45 ml/min per 1.73 m^2^ at long-term follow-up. There were 64 of 1475 patients with tumor with follow-up eGFR testing over a median 4.3 years that developed progressive CKD and 37 that developed kidney failure. All measures of larger nephron size and nephrosclerosis were associated with both outcomes and all except artery luminal stenosis remained significant after adjustment for clinical characteristics (Table 5).

**Table 4:** Chronic changes derived by AI as predictors of short-term (median 4.4 mos) measured GFR < 45 ml/min per 1.73 m^2^ among 1166 living kidney donors (42 events), and long-term (median 10.5 years) estimated GFR < 45 ml/min per 1.73 m^2^ among 671 living kidney donors (32 events)

Per SD	Short-term measured GFR < 45 ml/min per 1.73 m^2^				Long-term estimated GFR < 45 ml/min per 1.73 m^2^			
	Unadjusted		Adjusted^a^		Unadjusted		Adjusted^a^	
	OR (95% CI)	*P* value	OR (95% CI)	*P* value	OR (95% CI)	*P* value	OR (95% CI)	*P* value
Nephron size								
Glomerular volume	0.86 (0.61–1.18)	0.38	1.01 (0.71–1.39)	0.95	1.48 (1.06–2.07)	0.02	1.63 (1.12–2.36)	0.009
Cortex per glomerulus	1.12 (0.85–1.36)	0.33	1.22 (0.89–1.59)	0.18	1.72 (1.19–2.47)	0.004	1.60 (1.09–2.34)	0.01
Mean proximal tubular area	1.13 (0.83–1.51)	0.42	1.38 (1.01–1.87)	0.04	1.11 (0.78–1.55)	0.56	1.18 (0.80–1.73)	0.38
Mean distal tubular area	1.14 (0.85–1.49)	0.30	1.21 (0.88–1.63)	0.23	1.19 (0.83–1.68)	0.33	1.16 (0.78–1.70)	0.46
Nephrosclerosis								
%GSG	1.70 (1.39–2.05)	< 0.001	1.37 (1.09–1.70)	0.005	1.42 (1.11–1.82)	0.006	1.19 (0.89–1.54)	0.22
%TA or %ITA								
TA area per tubular area, %	1.18 (1.00–1.40)	0.046	0.91 (0.68–1.10)	0.42	1.31 (1.04–1.65)	0.02	1.12 (0.82–1.45)	0.42
Interstitium and TA area per tubulointerstitial area, %	1.10 (0.81–1.46)	0.51	0.87 (0.61–1.21)	0.42	1.39 (1.01–1.92)	0.04	1.20 (0.83–1.69)	0.32
TA foci density								
TA foci density, per tubular area	1.45 (1.19–1.76)	< 0.001	1.02 (0.80–1.27)	0.85	1.35 (1.06–1.67)	0.008	1.13 (0.83–1.45)	0.38
Vascular changes								
AH density, per cortex area	1.20 (0.91–1.51)	0.15	1.18 (0.83–1.51)	0.22	1.32 (1.04–1.67)	0.02	1.26 (0.94–1.62)	0.09
%Arterioles with AH	1.24 (0.97–1.53)	0.06	1.22 (0.93–1.55)	0.11	1.35 (1.02–1.80)	0.04	1.17 (0.84–1.57)	0.33
Mean AH area	1.15 (0.86-1.45)	0.30	1.15 (0.85-1.49)	0.32	1.61 (1.21–2.13)	0.001	1.54 (1.12-2.08)	0.006
%Luminal stenosis of arteries	1.29 (0.95–1.69)	0.09	1.03 (0.73–1.42)	0.87	1.41 (1.04–1.91)	0.02	1.36 (0.96–1.88)	0.07

AH, arteriolar hyalinosis; CI, confidence interval; GFR, glomerular filtration rate; GSG, globally sclerosed glomeruli; IFTA, interstitial fibrosis and tubular atrophy; ITA, interstitium and tubular atrophy; OR, odds ratio; TA, tubular atrophy.

a Adjusted for age, sex, baseline estimated GFR, and follow-up time.

**Table 5:** Chronic changes derived by AI as predictors of progressive CKD (64 events) or ESKD (37 events) among 1475 patients with a renal tumor that had no death, cancer recurrence or started dialysis during the first 4 months following radical nephrectomy

Per SD	Progressive CKD				Kidney failure			
	Unadjusted		Adjusted^a^		Unadjusted		Adjusted^a^	
	HR (95% CI)	*P* value	HR (95% CI)	*P* value	HR (95% CI)	*P* value	HR (95% CI)	*P* value
Nephron size								
Glomerular volume	1.82 (1.54–2.14)	< 0.001	1.74 (1.40–2.16)	< 0.001	1.73 (1.38–2.18)	< 0.001	1.42 (1.07–1.90)	0.02
Cortex per glomerulus	1.81 (1.64–2.01)	< 0.001	1.59 (1.30–1.95)	< 0.001	1.83 (1.62–2.06)	< 0.001	1.34 (1.09–1.65)	0.006
Mean proximal tubular area	1.81 (1.45–2.24)	< 0.001	1.67 (1.29–2.16)	< 0.001	1.92 (1.46–2.54)	< 0.001	1.57 (1.11–2.21)	0.01
Mean distal tubular area	1.80 (1.46–2.23)	< 0.001	1.79 (1.40–2.28)	< 0.001	1.81 (1.38–2.38)	< 0.001	1.75 (1.26–2.44)	0.0009
Nephrosclerosis								
%GSG	1.99 (1.75–2.27)	< 0.001	1.63 (1.37–1.95)	< 0.001	2.11 (1.81–2.47)	< 0.001	1.55 (1.23–1.94)	< 0.001
%TA, or %ITA								
TA area per tubular area, %	1.32 (1.17–1.49)	< 0.001	1.28 (1.07–1.51)	0.006	1.38 (1.21–1.58)	< 0.001	1.30 (1.02–1.66)	0.03
Interstitium and TA area per tubulointerstitial area, %	1.46 (1.22–1.74)	< 0.001	1.36 (1.11–1.68)	0.003	1.51 (1.20–1.88)	< 0.001	1.34 (1.01–1.78)	0.04
TA foci density								
TA foci density, per tubular area	1.75 (1.51–2.04)	< 0.001	1.59 (1.31–1.93)	< 0.001	1.85 (1.54–2.23)	< 0.001	1.60 (1.21–2.10)	< 0.001
Vascular changes								
AH density, per cortex area	1.72 (1.44–2.06)	< 0.001	1.49 (1.23–1.80)	< 0.001	1.71 (1.36–2.15)	< 0.001	1.36 (1.06–1.73)	0.02
%Arterioles with AH	1.57 (1.33–1.89)	< 0.001	1.40 (1.17–1.67)	< 0.001	1.48 (1.19–1.84)	< 0.001	1.28 (1.01–1.63)	0.04
Mean AH area	1.71 (1.43–2.04)	< 0.001	1.54 (1.26–1.88)	< 0.001	1.76 (1.40–2.20)	< 0.001	1.47 (1.12–1.94)	0.005
%Luminal stenosis of arteries	1.45 (1.15–1.82)	0.002	1.19 (0.92–1.55)	0.19	1.59 (1.19–2.12)	0.002	1.32 (0.93–1.87)	0.12

AH, arteriolar hyalinosis; CI, confidence interval; GSG, globally sclerosed glomeruli; HR, hazard ratio; IFTA, interstitial fibrosis and tubular atrophy; ITA, interstitium and tubular atrophy; TA, tubular atrophy.

a Adjusted for age, sex, body mass index, hypertension, diabetes, baseline eGFR, and proteinuria.

### Chronicity Scores in Kidney Patients With Tumor

**Figure 5:**
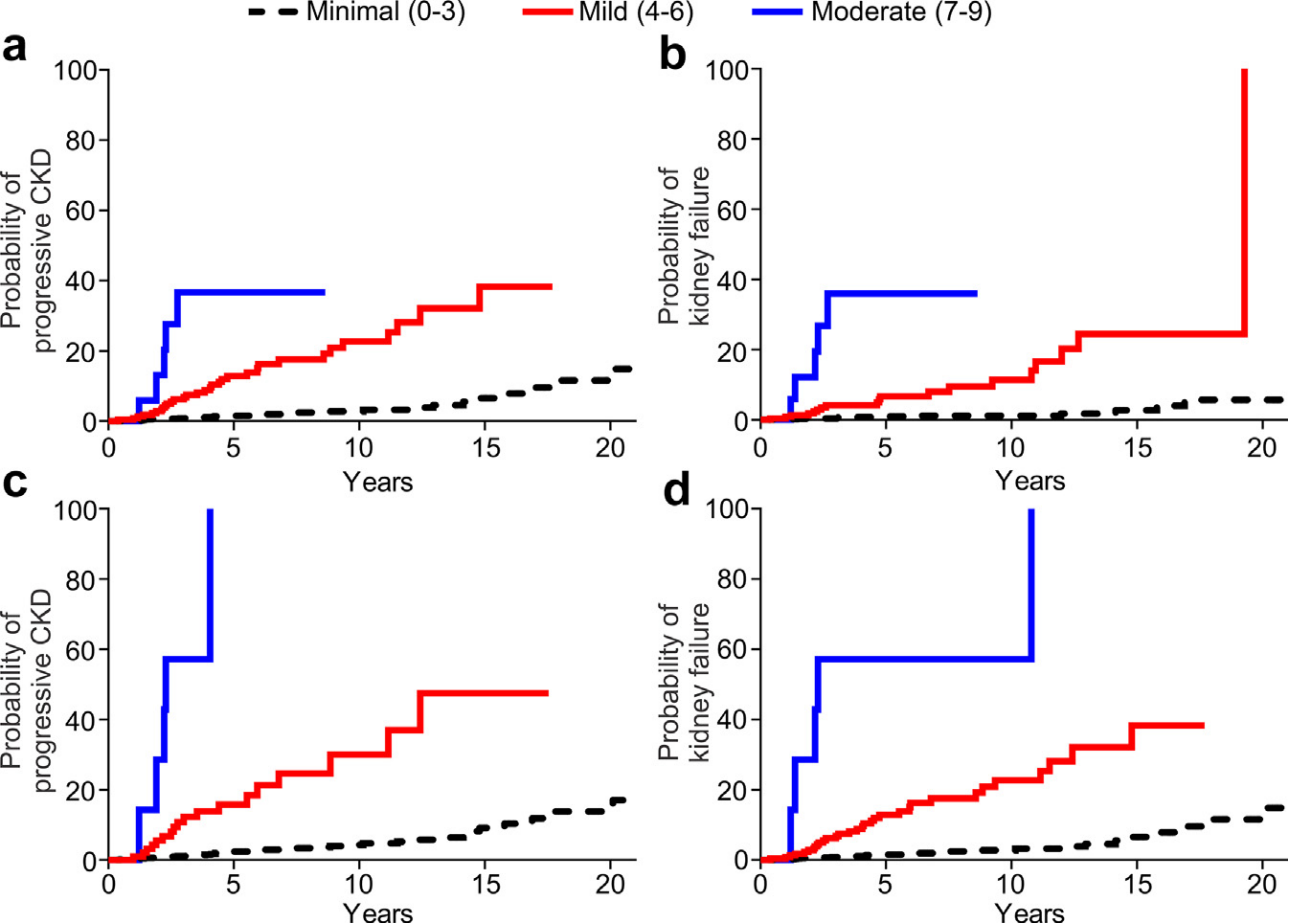
Risk of progressive CKD and kidney failure from 4 months postnephrectomy based on 2 chronicity scores. Risk of progressive CKD and kidney failure based on severity of Nephrosclerosis Chronicity Score (a and b), Nephron Hypertrophy and Nephrosclerosis Chronicity Score (c and d). Nephrosclerosis Chronicity Score was obtained by summing the %GSG score (0–3), %ITA score (0–3), TA density score (0–3), and AH area score (0–3). Nephron Hypertrophy and Nephrosclerosis Chronicity Score was obtained by summing the cortex per glomerulus score (0–3), %GSG score (0–3), TA density score (0–3), and AH area score (0–3). Each of the 4 variables (0–3) was summed, but the highest chronicity score among these patients was 9 (not 12). The chronicity scores (0–9) were displayed across three levels (0–3, 4–6, and 7–9). %GSG, percent globally sclerosed glomeruli; AH, arteriolar hyalinosis; CKD, chronic kidney disease; %ITA, percent interstitium and TA cluster; TA, tubular atrophy.

We created 2 chronicity scores and evaluated their performance in the patients with a renal tumor. The Nephrosclerosis Chronicity Score was based on our previously published score we developed in patients with native kidney disease using percentage of globally sclerosed glomeruli, %ITA, TA foci density, and mean AH area. We derived a separate Nephron Hypertrophy and Nephrosclerosis Chronicity Score using LASSO to select from among all the nephron size and nephrosclerosis measures, the 4 measures that best predicted progressive CKD in multivariable analysis. These 4 variables chosen were identical to those in our Nephrosclerosis Chronicity Score except that %ITA was replaced by cortex per glomerulus. To make 0 to 12 point scores, percent globally sclerosed glomeruli, and %ITA per tubulointerstitium were converted into scores of 0 to 3 based on the commonly used thresholds (< 10%, 10%–25%, 26%–50%, > 50%).^11^ TA density per tubular area, AH area, and cortex per glomerulus were converted into a 0 to 3 score using *a priori* chosen thresholds, respectively (TA density, 0: ≤ 5, 1: 6–10, 2: 11–20, and 3: > 20 foci per mm^2^; AH area, 0: ≤ 500, 1: 501–750, 2: 751–1 000, and 3: > 1000 μm^2^; and cortex per glomerulus, 0: ≤ 0.075, 1: 0.076–0.125, 2: 0.126–0.175, and 3: > 0.175 mm^3^). The Nephrosclerosis Chronicity Score (0–12) was created by summing percent globally sclerosed glomeruli score (0–3), %ITA score (0–3), TA density score (0–3), and AH area score (0–3). The Nephron Hypertrophy and Nephrosclerosis Chronicity Score was the same except that it replaced the %ITA score (0–3) with the cortex per glomerulus score (0–3). The highest score was a 9 for both chronicity scores. The Nephron Hypertrophy and Nephrosclerosis Chronicity Score better predicted progressive CKD than the Nephrosclerosis Chronicity Score (Table 6, Figure 5).

**Table 6:** Risk of CKD and ESKD in patients with tumor per 1-point increase in renal chronicity scores

Chronicity scores	Progressive CKD				Kidney failure			
	Unadjusted		Adjusted for clinical characteristics^a^		Unadjusted		Adjusted for clinical characteristics^a^	
	HR	*P* value	HR	*P* value	HR	*P* value	HR	*P* value
Nephrosclerosis Chronicity score^b^ [%GSG score (0–3) + %ITA score (0–3) + TA foci density score (0–3) + AH area score (0–3)]	1.91 (1.67–2.19)	< 0.001	1.66 (1.42–1.94)	< 0.001	2.17 (1.81–2.60)	< 0.001	1.69 (1.36–2.10)	< 0.001
C-statistic^c^	0.792/0.778		0.811/0.795		0.824/0.811		0.850/0.835	
Nephron Hypertrophy and Nephrosclerosis Chronicity score^d^ [Cortex per glomerulus score (0–3) + %GSG score (0–3) + TA foci density score (0–3) + AH area score (0–3)]	2.07 (1.83–2.34)	< 0.001	1.84 (1.57–2.16)	< 0.001	2.20 (1.89–2.56)	< 0.001	1.72 (1.40–2.12)	< 0.001
C-statistic^c^	0.816/0.803		0.840/0.829		0.827/0.819		0.843/0.829	

AH, arteriolar hyalinosis; CI, confidence interval; ESKD, end-stage kidney disease; GSG, globally sclerosed glomeruli; HR, hazard ratio; ITA, interstitium and tubular atrophy; TA, tubular atrophy.

All AI measures were first converted into scores.

Shown are HRs (95% CIs) per 1-point increase in the score (range from 0–12).

a Adjusted for age, sex, hypertension, diabetes, body mass index, eGFR and proteinuria.

b Nephrosclerosis Chronicity Score was generated by sum of the %GSG score (0–3), %ITA score (0–3), TA foci density score (0–3), and AH area score (0–3).

c Second value is C-statistic after 10-fold cross-validation. The Nephron Hypertrophy and Nephrosclerosis Chronicity Score better discriminated progressive CKD outcomes than the Nephrosclerosis Chronicity score (*P* < 0.05 with or without adjustment for clinical characteristics). The 2 scores performed similarly for the kidney failure outcome.

d Nephron Hypertrophy and Nephrosclerosis Chronicity Score was generated by sum of the cortex per glomerulus score (0–3), %GSG score (0–3), TA foci density score (0–3), and AH area score (0–3).

## Discussion

In this study, we developed a multiclass AI model that detects the major microstructures and chronic pathology on WSIs of kidney histology. We then used the AI model detections, accounting for edge effects, to automate quantification of different measures of nephron size and nephrosclerosis. We further showed that automated measures of enlarged nephron size and nephrosclerosis were individually associated with CKD outcomes and could be combined into 12-point prognostic scores. These nephron size and nephrosclerosis measures based on AI model detections associated with clinical characteristics and CKD outcomes in a pattern similar to previous reports using simplified manual annotations in these WSIs.^3^^,^^4^^,^^6^^,^^7^ In addition, there were several novel findings because of the use of AI detections rather than manual annotations. The AI model detections could be used to calculate the mean cross-sectional areas of proximal and distal tubules, which predicted CKD outcomes. The AI model allowed separate detection of interstitium from TA and a more detailed characterization of AH, both of which predicted CKD outcomes. The AI model also allowed us to quantify TA per tubular area rather than per cortex area and to quantify interstitium and TA per tubulointerstitial area rather than per cortex area. Notably, both TA foci density and mean AH area were important predictors in the chronicity scores and both would be difficult to score with human vision.

Studies in living kidney donors and patients with a renal tumor have shown that measures of nephron size and nephrosclerosis associated with future CKD outcomes,^1^^,^^3^^,^^4^^,^^6^^,^^7^^,^^27^, ^28^, ^29^ or with graft loss in kidney transplant recipients.^19^ In clinical practice, semiquantitative estimates by human vision are reported by pathologists, despite known limitations in accuracy and precision.^9^ Given the labor-intensive approach of manual annotating and tracing structures on kidney histology, an automated approach is needed to make quantitative measures feasible. Clinical associations with AI measures of nephron size and nephrosclerosis showed a similar patten between donors and patients with tumor. The increased variability and severity of chronic changes in the patients with a renal tumor allowed us to develop and evaluate chronicity scores similar to those developed in patients with native kidney disease,^2^^,^^5^^,^^11^ but based on AI model detections rather than human annotations.

For several structures used to estimate chronic changes, AI had specific advantages where a manual approach would be more challenging. Distinguishing the exact boundary between cortex and medulla is challenging by visual inspection, particularly on a needle biopsy section. By training our AI model with large wedge sections rather than just needle biopsy sections, we could train the model with better ground truth for cortex versus medulla. In addition, the AI model allowed quantification of the area of interstitium, and separately, the mean cross-sectional areas of proximal and distal tubules. The interstitium between tubules is difficult to approximate by human vision and by manual morphometry would require tedious annotation of every tubule, vessel, and glomerulus.

The AI model comprehensively quantified AH lesions within arterioles, which would have been impractical to do with manual annotations, particularly on wedge sections. Importantly, AH was associated with CKD outcomes in both living kidney donors and patients with a renal tumor, even after adjusting for clinical characteristics. Further, both the mean area of AH lesions and the density of AH lesions (per cortex area or per arteriole) were associated with CKD outcomes, though the mean area of AH lesions was the more prognostic predictor and was incorporated into the chronicity scores. Indeed, AH in arterioles was a more prognostic predictor of CKD outcomes than was luminal stenosis in arteries from intimal thickening. Past studies have found AH by visual inspection to be associated with older age, hypertension, and lower eGFR^30^^,^^31^; and to predict subsequent decline in eGFR or kidney failure.^2^^,^^10^^,^^32^^,^^33^ Our study extends these findings, showing that an AI model can detect even subtle amounts of AH and characterize the lesions by both their density and size to detect an increased risk of CKD outcomes, even in living kidney donors.

This study has several limitations to consider. The clinical evaluation of renal biopsy tissue specimens often includes multiple tissue section levels. This study only assessed a single PAS-stained section because certain chronic changes such as AH are best detected on PAS-stained sections. It is possible to convert images of kidney tissue in other stains (e.g., hematoxylin and eosin) to a PAS-stained image.^34^ Alternatively, a model could be trained specifically using images of sections with other stains. The study was limited to living kidney donors and patients with a renal tumor. Future studies are needed to optimize and test this models performance in patients undergoing kidney biopsy specifically for parenchymal kidney disease.

In conclusion, this study created a multiclass AI-based segmentation model that detects structures that can be used to quantify chronic changes from a WSI of a PAS-stained kidney tissue section in a standardized and reproducible manner. Converting this or similar AI models into a clinical tool has the potential to improve efficiency, reproducibility, quantification of chronic structural changes; and enhance the value of pathology reports. Such a clinical tool would generate a quantitative report of nephron size and nephrosclerosis measures along with reference ranges based on normotensive living kidney donors as previously done with human annotations.^1^

## Disclosure

All the authors declared no competing interests.

## Supplementary Material

Supplementary File (PDF) **Supplementary Methods. Supplementary References. Figures S1.** Examples of training regions and annotations in the tissue layer in both wedge sections and a needle biopsy. **Figures S2.** Examples of training regions and annotations in the three tissue sublayers in both wedge sections and a needle biopsy. **Figure S3.** Examples of training regions and annotations in all remaining cortex sublayers. **Table S1.** Calculations of chronic changes from AI detections. **Table S2.** Correlations between chronic changes and clinical characteristics.

## Footnotes

Supplementary File (PDF)

**Supplementary Methods.**

**Supplementary References.**
**Figures S1.** Examples of training regions and annotations in the tissue layer in both wedge sections and a needle biopsy. **Figures S2.** Examples of training regions and annotations in the three tissue sublayers in both wedge sections and a needle biopsy. **Figure S3.** Examples of training regions and annotations in all remaining cortex sublayers. **Table S1.** Calculations of chronic changes from AI detections. **Table S2.** Correlations between chronic changes and clinical characteristics.
