# Supplementary material for: Chronic Changes on Kidney Histology by a Multiclass Artificial Intelligence Model
Source: Kidney Int Rep. 2025 May 29;10(8):2668–79. doi: 10.1016/j.ekir.2025.05.035 (PMC12347913; doi:10.1016/j.ekir.2025.05.035)
Supplement: Supplementary File (PDF) — Supplementary Methods. Supplementary References. Figures S1. Examples of training regions and annotations in the tissue layer in both wedge sections and a needle biopsy. Figures S2. Examples of training regions and annotations in the three tissue sublayers in both wedge sections and a needle biopsy. Figure S3. Examples of training regions and annotations in all remaining cortex sublayers. Table S1. Calculations of chronic changes from AI detections. Table S2. Correlations between chronic changes and clinical characteristics. [file mmc1.pdf]

Supplementary Material

1. Supplementary Methods

AI model iterative training

The model was developed using Aiforia Create (Aiforia Technologies, Helsinki, Finland), a cloud-based, supervised deep learning platform designed for analyzing tissue histology images. It allows users to annotate examples of different classes into a customizable layer tree followed by training convolutional neural networks for segmentation or object detection with instance segmentation based on the annotations.[S1-3] We initially selected 100 images from kidney donors and 100 images from kidney tumor patients (one image per patient) using HistoQC to ensure training on a wide spectrum of images,[14] but 7 were subsequently excluded due to severe fading of the stain. Hence, the model was developed on the remaining 193 images (94 living kidney donor biopsies and 99 wedge sections from tumor patients). The ground truth annotations were performed by an experienced morphometrist (AD) and research fellows (MK and MSA) with quality control checks of their work by AD. Training and guidance throughout the study was provided by renal pathologists (MPA, MLS, FES and LB). The training of the model on the 193 whole slide images resulted in a layered multiclass model (a set of nested individual convolutional neural networks). Each layer of the model was designed to detect a single class of kidney structures from background or multiple mutually exclusive classes from background. Ground truth annotations were provided via the Aiforia Create interface to identify each class within a layer. Both semantic and instance segmentation classes were created. The ground truth annotations were developed across one or more individual regions within the image and included training annotations for both "true positives" and "true negatives" for each class as training annotations between competing classes and their boundaries. The process of training the AI model with ground truth was iterative. A layer was trained based on the current set of ground truth annotations and then assessed for its performance. With iterative guidance and input from renal pathologists, this allowed efficient correction of "false positives" to be annotated as ground truth examples of "true negatives" and "false negatives" to be annotated as ground truth examples of "true positives". For example, if the AI model was detecting tubular casts as globally sclerotic glomeruli, we would annotate these tubular casts as ground truth for what were not globally sclerotic glomeruli. Likewise, if the AI model was missing small obsolescent globally sclerotic glomeruli, we would annotate these missed glomeruli as ground truth for what were globally sclerotic glomeruli.

AI model training strategy for each class

The final model had 20 classes across 8 nested layers (**Figure 2**). The first layer had one class TISSUE. Within this class, cortex, medulla, kidney capsule and vessel lumens were trained as positive, but internal tissue tears were trained as negative. The next layer was under TISSUE and had 3 classes: CORTEX, MEDULLA, and INTERLOBAR ARTERIES (included arcuate artery). The wedge section images from kidney tumor patients were particularly advantageous for training this layer to distinguish and define boundaries between cortex and medulla. INTERLOBAR ARTERIES were defined as a separate class in this as this helped decrease misclassification between cortex and medulla. The CORTEX layer had 3 additional classes: ARTERIES AND ARTERIOLES, TUBULAR ATROPHY CLUSTER, and CORTEX EXCLUDING ARTERIES AND ATROPHIC TUBULES. Large foci of inflammation without tubules or atrophic tubules were not trained into a class in this layer and become part of the background cortex. Under ARTERIES AND ARTERIOLES, 3 additional classes were trained: ARTERIAL LUMEN, INTIMAL THICKENING, and ARTERIOLAR HYALINOSIS. The ARTERIAL LUMEN class was trained in arteries and not in arterioles, and thus presence of this ARTERIAL LUMEN class was used to distinguish arteries from arterioles. Similar to the interlobar arteries, intima in cortical intralobular arteries that was not thickened was not a class in the model. Arteriolar

hyalinosis was only trained in arterioles. The TUBULAR ATROPHY CLUSTER class was trained with regions of singleton or clusters of atrophic tubules that were mature (atrophy of the tubule had to be present not simply thickening of tubular basement membrane). Interstitial fibrosis between but not surrounding clusters of atrophic tubules was not included in the TUBULAR ATROPHY CLUSTER class as this led to a model class with fewer false positives and false negatives. A layer under CORTEX EXCLUDING ARTERIES AND ATROPHIC TUBULES had two classes: GLOMERULAR AREA and TUBULOINTERSTITIUM. The GLOMERULAR AREA class was defined using the glomerular capsule or outer boundary of a globally sclerotic glomeruli if a capsule was no longer evident. Under the TUBULOINTERSTITIUM class we trained a TUBULE class to detect tubules, venules, and lymphatics (but not peritubular capillaries). The background for the TUBULE class within the TUBULOINTERSTITIUM class defined interstitium. These aforementioned classes were all semantic segmentations. There were additional instance level segmentation classes under the GLOMERULAR AREA layer and the TUBULE layer. There were 3 objects classes under GLOMERULAR AREA layer: GLOBALLY SCLEROTIC GLOMERULI, NON-GLOBALLY SCLEROTIC GLOMERULI, and EMPTY CAPSULE. The 2 object classes under the TUBULE were PROXIMAL TUBULE and DISTAL TUBULE. Venules, lymphatics, and tubules that were not clearly proximal or distal, tubules with pathology were not trained as object classes under the TUBULE layer. Representative examples of how each layer and class were trained are shown in **Figures S1-3**.

Validation of AI model within Aiforia create

The accuracy of the full final AI segmentation model was then compared to the human segmentation in an independent “test set” of whole slide images from 5 kidney tumor patient and 5 living kidney donor kidney biopsy images using the Analytical Validation tool within Aiforia Create that compares interobserver variability between humans to that of AI with humans. Seven pathologists (each with training and expertise in identifying chronic changes on renal pathology) annotated within assigned regions the classes for each layer across all 10 images. There were 390 object class regions and 220 semantic class regions among all 20 classes annotated across the 10 images by each of the 7 pathologists. From the classes independently identified by each human segmentation and AI segmentation model, inter-operator agreement statistics between both AI vs human and human vs human were calculated. There was separate comparison of all humans’ averaged versus AI (Human versus AI) and the average between each human pair (Human versus Human). These statistics compared were % false positive, % false negative, % total error (sum of false positive and false negative), precision, sensitivity, and F1 score. Precision is defined as the percentage of the analysis findings that overlap with annotated object, calculated of all analysis results in the validation regions. Sensitivity is defined as the percentage of all annotations (counts or area) that were found by the analysis, calculated of all annotations in the validation regions. F1 score is the harmonic mean of precision and sensitivity. **Table 1** shows the results of the validation for all 20 classes. The F1 score was similar between AI versus Human and Human versus Human for all classes except for PROXIMAL TUBULE, DISTAL TUBULE, and ARTERIOLAR HYALINOSIS that were lower by AI versus Human than by Human versus Human. The F1 scores were above 90% in most classes. They were lower with ARTERIOLAR HYALINOSIS for AI versus Human (64.1%) than Human versus Human (79.8%). They were below 90% but similar performance between AI versus human to that of human versus human for TUBULAR ATROPHY CLUSTER (84.6% and 85.6%) and INTIMAL THICKENING (87.6% and 86.3%) of INTERLOBAR ARTERIES.

Nephron size and nephrosclerosis morphometry from AI model detection of structure classes

The final AI model was applied to all PAS-stained whole slide images for 1426 living kidney donors and 1699 tumor patient in the AKA study. This took 5 to 12 minutes per image depending on usage of the Cloud environment by other investigators. There was no additional human review or modification of the AI model

detections. From the detection of each class structure in each image, the count of each non-contiguous structure for each class (including for semantic classes) and the total area of that class was calculated by the AIFORIA software. Edge effects due to bisection of structures by the biopsy needle need to be accounted for in the morphometric analysis with needle core biopsy sections.[S4-6] Thus for the NON-GLOBALLY SCLEROTIC GLOMERULI, EMPTY CAPSULE, TUBULAR ATROPHY CLUSTER, and ARTERY AND ARTERIOLES classes we obtained separate counts and areas for those adjacent to the CORTEX border and non-tissue background using spatial metrics tool within Aiforia Create. Notably, this only identify objects adjacent to an edge bisected by the biopsy needle and not adjacent to kidney capsule or medulla, since we used the CORTEX boundary rather than tissue boundary with non-tissue background. Since ARTERIOLAR HYALINOSIS can occur as 2 separate lesions on a single arteriole, or a single lesion on 2 adjacent arterioles, we used a spatial metric that identifies and counts only ARTERIOLAR HYALINOSIS lesions at least 500μm apart from each other to count only one ARTERIOLAR HYALINOSIS lesion within a single arteriole profile.

All NON-GLOBALLY SCLEROTIC GLOMERULI and EMPTY CAPSULE bisected at edge of the cortex and non-tissue background in needle biopsies were counted as 0.675.[26] From these AI detected structural counts (corrected for the edge effects) and areas we calculated different measures of nephron size and nephrosclerosis. Using Weibel and Gomez stereology models we calculated glomerular volume and cortex per glomerulus (inverse of volumetric glomerular density).[S7] We calculated %GSG from the counts of GLOBALLY SCEROTIC GLOMERULI divided by the counts of all glomeruli (GLOBALLY SCEROTIC GLOMERULI, NON-GLOBALLY SCLEROTIC GLOMERULI and EMPTY CAPSULE). By dividing the areas of PROXIMAL and DISTAL TUBULES by their count we obtained mean proximal and distal tubular areas. Interstitium area was determined by subtracting the TUBULES area from the Tubulointerstitium area. TUBULAR ATROPHY CLUSTER that were adjacent to the edge of the cortex and non-tissue background were counted as 0.5.[1] We obtained %TA and TA foci density of tubular area by dividing the area or counts of TUBULAR ATROPHY CLUSTER by the sum of TUBULES area and TUBULE ATROPHY CLUSTER area. We obtained %interstitium and TA of tubulointerstitium by dividing the areas of interstitium and TUBULE ATROPHY CLUSTER by the areas of TUBULOINTERSTITIUM and TUBULE ATROPHY CLUSTER. The number of ARTERIOLAR HYALINOSIS lesions was divided by the CORTEX area to calculate the arteriolar hyalinosis density per cortex area. We calculated % arterioles with arteriolar hyalinosis by dividing the number of arterioles with arteriolar hyalinosis by the total number of arterioles. Finally, we calculated mean arteriolar hyalinosis area by dividing the total area of ARTERIOLAR HYALINOSIS by the count of ARTERIOLAR HYALINOSIS. Cases with no ARTEROLAR HYALINOSIS were assigned to have a mean arteriolar hyalinosis area of 0. Using INTIMAL THICKENING areas and ARTERIAL LUMEN areas we estimated %luminal stenosis (narrowing of lumen due to arteriosclerosis) as previously reported.[S4, 5] We calculated mean %luminal stenosis among all arteries with a lumen that was not adjacent to the cortex boundary (could be bisected by needle) and had an area >7850μm^2 (an area corresponds to a diameter of 100μm, a cutoff used to distinguish arterioles from arteries).[S8]

2. Supplementary Figures

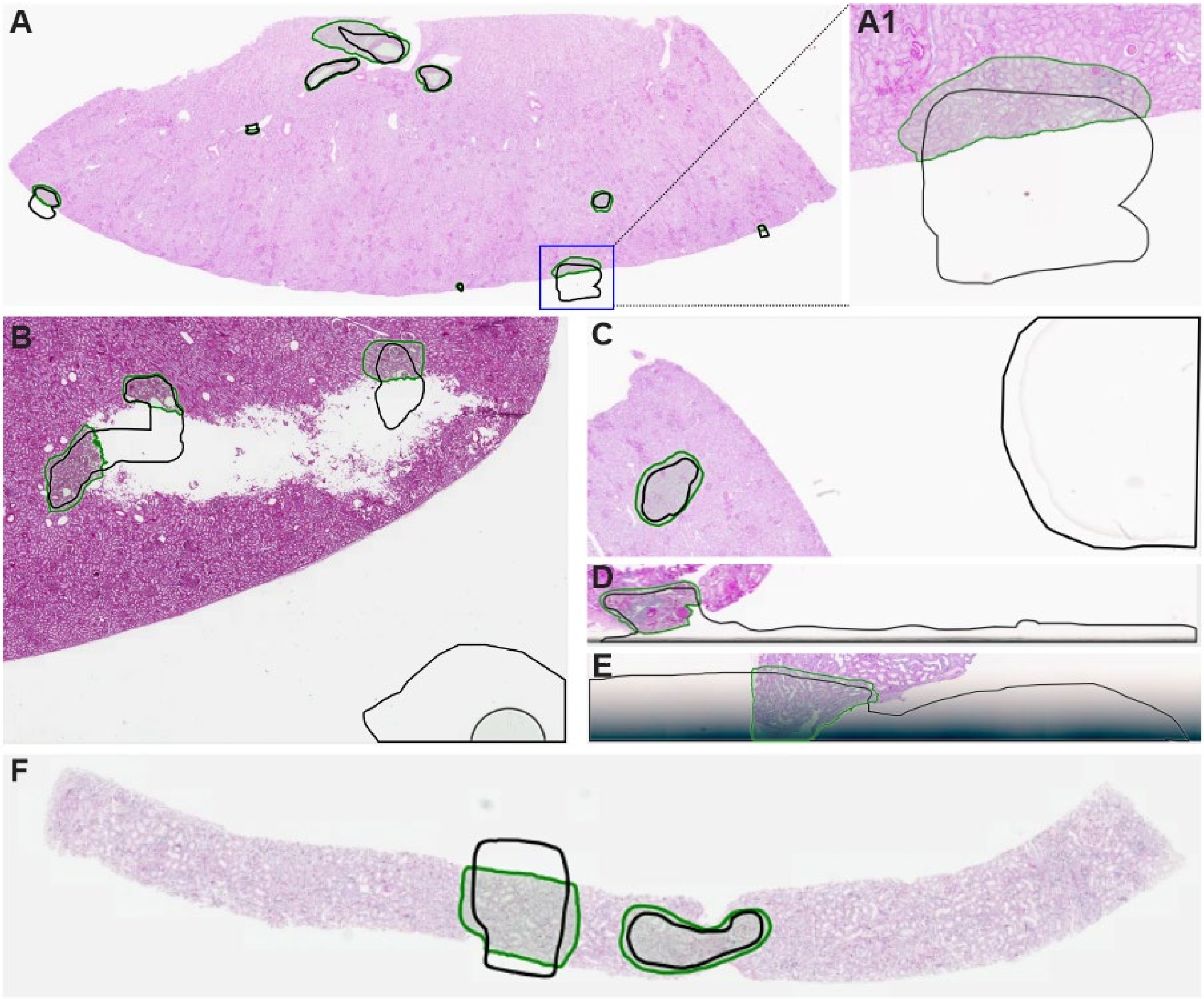

Figure S1. **Examples of training regions and annotations in the tissue layer in both wedge sections and a needle biopsy**. **A**) An example of a wedge section with several training regions of interest (black traces) and training annotations (green traces). The model is trained to recognize tissue if there is an overlap between training ROI and training annotation. **A1** shows a closer look where the training annotation is closely following the tissue contour, and the absence of overlap trains the model as a background. **B**) An example of a different wedge section with a tissue tear artifact, and the approach used to train the hole as a background. **C-E**) Various examples of artifacts on a glass slide that were trained as a background. **F**) An example of a similar approach to train a tissue layer in a needle biopsy.

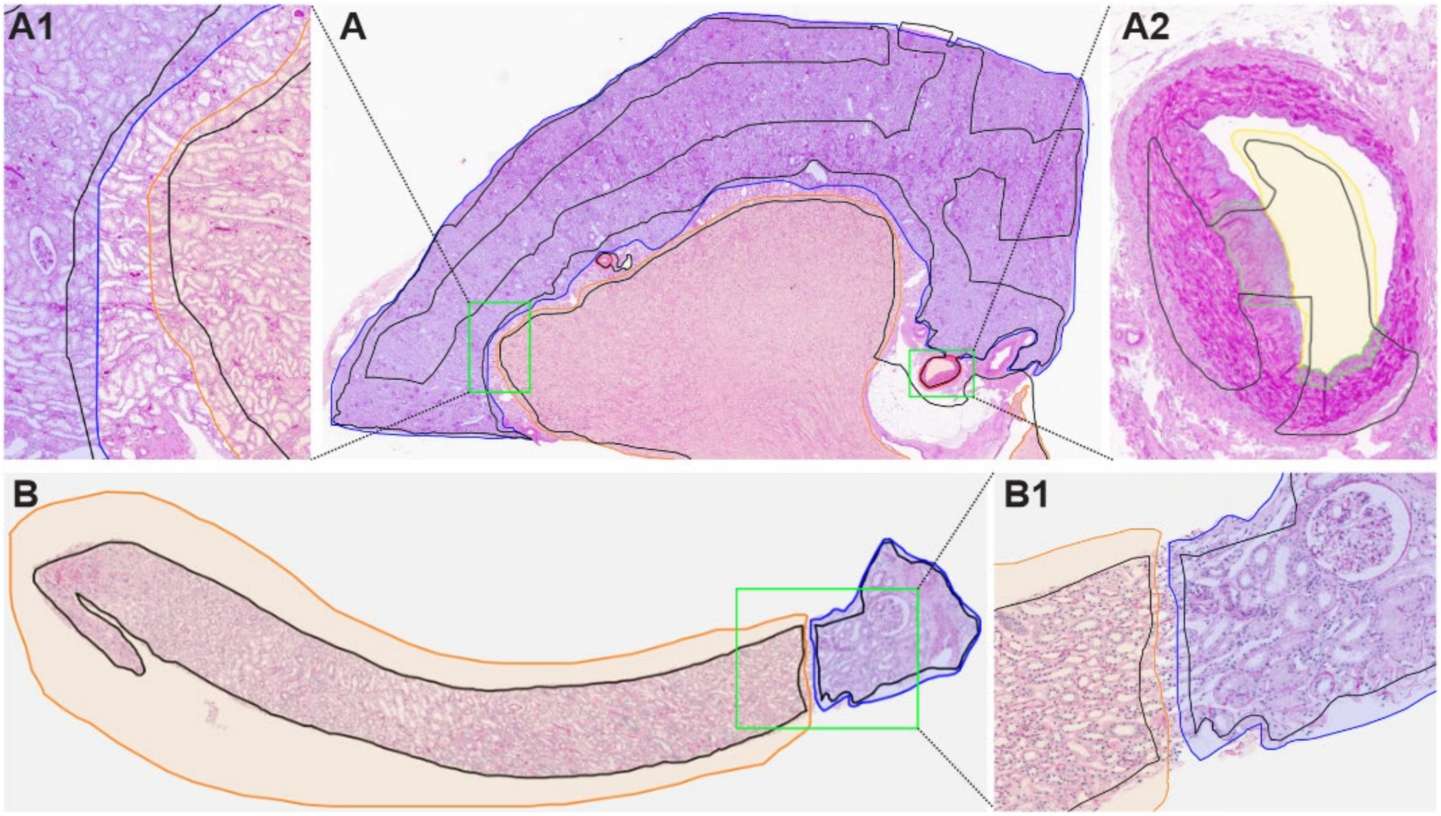

Figure S2. **Examples of training regions and annotations in the three tissue sublayers in both wedge sections and a needle biopsy**. **A**) An example of a wedge section showing the training annotations for 3 classes, cortex (blue), medulla (orange) and interlobar arteries (red). **A1** shows a closer look of the approach; a gap between training ROIs for cortex and medulla was left to avoid human-related bias in separating cortex from medulla. **A2** shows a closer look at the interlobar artery and how the model was trained to recognize intima (light green) and lumen (yellow), but not the media layer. **B)** An example of a similar approach to train cortex and medulla in a needle biopsy, with a closer look shown in **B1**. Arcuate arteries were usually absent from the needle biopsies.

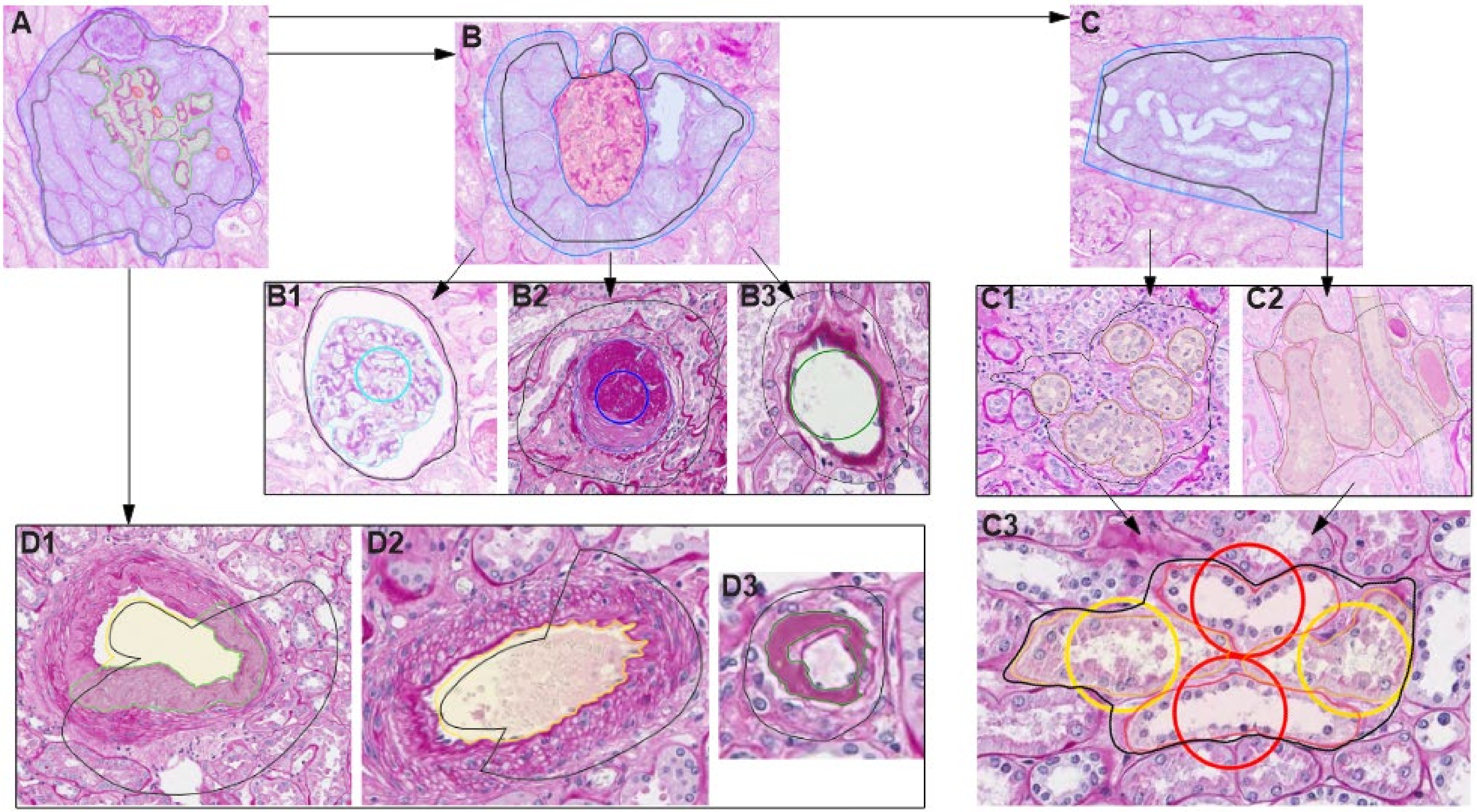

Figure S3. **Examples of training regions and annotations in all remaining cortex sublayers**. **A**) An example shows a training region and annotations how cortex was subdivided into three regions: cortex excluding arteries and atrophic tubules (dark blue), tubular atrophy cluster (green) and arteries and arterioles (red). **B-C**) Examples of how cortex excluding arteries and atrophic tubules is divided into glomerular area (red) and tubulointerstitium (blue). Under glomerular area model was trained as object and instance level segmentation to recognize **B1**) non-globally sclerosed glomerulus, **B2**) globally sclerosed glomerulus and **B3**) empty capsule. Under tubulointerstitium layer, a single tubule layer (light brown) was trained by providing precise annotations following tubular basement membrane. Two examples show approaches used in case of increased interstitial space (**C1**) or more densely packed tubules **(C2**). Regardless of the approach, in the final layer, proximal tubules (yellow) and distal tubules (red) were trained as objects and instance level segmentation (**C3**). If there was thickened intima, cortical arteries were trained similar to interlobar arteries (**D1**). If intima was not thickened, only lumen was trained (**D2**). In arterioles (**D3**), arteriolar hyalinosis (green) was traced when present.

3. Supplementary Tables

Table S1. Details on how measures of nephron size and nephrosclerosis were morphometrically calculated by AI.

**Measures of nephron size**	**Calculated from the AI model detections**
Glomerular volume	Calculated from NSG tuft profiles using Weibel-Gomez formula.[S7]
Cortex per glomerulus	Reciprocal of the 3-dimensional glomerular density calculated from NSG tuft profiles and cortex area using Weibel-Gomez formula.[S7] NSG profiles on edge counted as 0.675 profiles.[26]
Mean proximal tubular area	The total area of all detected proximal tubules divided by the number of proximal tubules
Mean distal tubular area	The total area of all detected distal tubules divided by the number of distal tubules
**Measures of nephrosclerosis**	
%GSG	Number of GSG profiles divided by number of NSG and GSG profiles. NSG profiles on edge counted as 0.675 profiles
%TA per tubular area	The area of TA divided by the area of tubules and TA
%ITA per tubulointerstitial area	The area of interstitium and TA divided by area of interstitium, tubules, and TA
TA density per tubular area	Number of TA divided by area of tubules and TA. TA foci on edge counted as 0.5 foci.[1]
AH density	Number of AH lesions (those within 500μm of each other were counted as 1) divided by area of cortex
% arterioles with AH	Number of arterioles with AH divided by the number of arterioles
Mean AH area, μm^2	The total area of all AH lesion divided by the number of AH lesions
%Luminal stenosis of arteries in cortex	The area of intima divided by the area of intima and lumen in all identified arteries (diameter >100μm)

NSG=non-globally sclerosed glomeruli, GSG=globally sclerosed glomeruli, IFTA=interstitial fibrosis and tubular atrophy, TA=tubular atrophy, ITA=interstitium and tubular atrophy, AH=arteriolar hyalinosis.

Table S2. Spearman's correlation of chronic changes derived by AI with clinical characteristics among 1426 living kidney donors and 1699 kidney tumor patients.

	**Age**		**Male**		**BMI**		**Hypertension**		**Diabetes**		**eGFR**		**[a]Proteinuria**	
	**LKD**	**TU**	**LKD**	**TU**	**LKD**	**TU**	**LKD**	**TU**	**LKD**	**TU**	**LKD**	**TU**	**LKD**	**TU**
**Nephron size**	**r_s (p value)**	**r_s (p value)**	**r_s (p value)**	**r_s (p value)**	**r_s (p value)**	**r_s (p value)**	**r_s (p value)**	**r_s (p value)**		**r_s (p value)**	**r_s (p value)**	**r_s (p value)**	**r_s (p value)**	**r_s (p value)**
Glomerular volume	**-0.05 (0.046)**	**-0.10 (<0.001)**	**0.16 (<0.001)**	**0.21 (<0.001)**	**0.26 (<0.001)**	**0.31 (<0.001)**	0.03 (0.22)	**0.15 (<0.001)**	..	**0.20 (<0.001)**	**0.07 (0.007)**	0.02 (0.37)	-0.04 (0.15)	**0.19 (<0.001)**
Cortex per glomerulus	0.04 (0.16)	**0.09 (<0.001)**	**0.10 (<0.001)**	**0.20 (<0.001)**	**0.20 (<0.001)**	**0.29 (<0.001)**	0.05 (0.07)	**0.23 (<0.001)**	..	**0.20 (<0.001)**	0.03 (0.22)	**-0.13 (<0.001)**	-0.04 (0.13)	**0.24 (<0.001)**
Mean proximal tubular area	-0.02 (0.46)	0.00 (0.96)	-0.02 (0.58)	**0.07 (0.008)**	**0.11 (<0.001)**	**0.27 (<0.001)**	**0.06 (0.02)**	**0.15 (<0.001)**	..	**0.17 (<0.001)**	**0.15 (<0.001)**	-0.01 (0.73)	**-0.13 (<0.001)**	**0.15 (<0.001)**
Mean distal tubular area	**0.05 (0.04)**	0.03 (0.20)	0.04 (0.10)	**0.17 (<0.001)**	**0.09 (<0.001)**	**0.21 (<0.001)**	**0.06 (0.03)**	**0.15 (<0.001)**	..	**0.13 (<0.001)**	0.03 (0.28)	**-0.08 (0.002)**	**-0.07 (0.005)**	**0.25 (<0.001)**
**Nephrosclerosis**														
%GSG	**0.26 (<0.001)**	**0.54 (<0.001)**	0.00 (0.92)	0.04 (0.07)	-0.01 (0.69)	-0.02 (0.45)	**0.12 (<0.001)**	**0.32 (<0.001)**	..	**0.11 (<0.001)**	**-0.18 (<0.001)**	**-0.42 (<0.001)**	0.02 (0.44)	**0.22 (<0.001)**
**%TA, or %ITA**														
TA area per tubular area, %	**0.45 (<0.001)**	**0.41 (<0.001)**	0.01 (0.74)	**0.13 (<0.001)**	0.01 (0.78)	0.01 (0.72)	**0.18 (<0.001)**	**0.31 (<0.001)**	..	**0.20 (<0.001)**	**-0.25 (<0.001)**	**-0.41 (<0.001)**	**0.07 (0.01)**	**0.25 (<0.001)**
Interstitium and TA area per tubulointerstitial area, %	**0.20 (<0.001)**	**0.20 (<0.001)**	**0.21 (<0.001)**	**0.23 (<0.001)**	**0.11 (<0.001)**	0.00 (0.84)	**0.10 (<0.001)**	**0.16 (<0.001)**	..	**0.10 (<0.001)**	**-0.10 (<0.001)**	**-0.30 (<0.001)**	0.01 (0.79)	**0.21 (<0.001)**
**TA foci density**														
TA foci density, per tubular area	**0.47 (<0.001)**	**0.42 (<0.001)**	0.02 (0.44)	**0.12 (<0.001)**	0.00 (0.88)	-0.02 (0.47)	**0.20 (<0.001)**	**0.30 (<0.001)**	..	**0.18 (<0.001)**	**-0.27 (<0.001)**	**-0.42 (<0.001)**	**0.07 (0.007)**	**0.25 (<0.001)**
**Vascular changes**														
AH density per cortex area	**0.11 (<0.001)**	**0.14 (<0.001)**	**0.10 (<0.001)**	**0.15 (<0.001)**	**0.06 (0.02)**	-0.01 (0.68)	**0.10 (<0.001)**	**0.15 (<0.001)**	..	**0.14 (<0.001)**	**-0.08 (0.003)**	**-0.17 (<0.001)**	**0.06 (0.03)**	**0.17 (<0.001)**
%Arterioles with AH	**0.11 (<0.001)**	**0.11 (<0.001)**	**0.10 (<0.001)**	**0.16 (<0.001)**	**0.06 (0.02)**	0.01 (0.64)	**0.10 (<0.001)**	**0.14 (<0.001)**	..	**0.16 (<0.001)**	**-0.08 (0.003)**	**-0.12 (<0.001)**	**0.06 (0.03)**	**0.15 (<0.001)**
Mean AH area	**0.10 (<0.001)**	**0.05 (0.04)**	**0.10 (<0.001)**	**0.13 (<0.001)**	**0.06 (0.02)**	**0.07 (0.003)**	**0.09 (<0.001)**	**0.14 (<0.001)**	..	**0.17 (<0.001)**	**-0.07 (0.01)**	**-0.09 (<0.001)**	0.03 (0.19)	**0.15 (<0.001)**
%Luminal stenosis of arteries	**0.22 (<0.001)**	**0.42 (<0.001)**	**-0.06 (0.02)**	**0.08 (<0.001)**	0.01 (0.63)	-0.02 (0.49)	0.04 (0.13)	**0.23 (<0.001)**	..	0.03 (0.22)	**-0.11 (<0.001)**	**-0.28 (<0.001)**	0.01 (0.66)	**0.18 (<0.001)**

LKD=living kidney donors, TU=Patients with tumor, GSG=globally sclerosed glomeruli, TA=tubular atrophy, ITA = interstitium and tubular atrophy, AH=arteriolar hyalinosis, BMI = body mass index, GFR = glomerular filtration rate.

[a]Proteinuria data available in 1421 donors and 1419 kidney tumor patients.

Bold values represent statistically significant results at $p<0.05$.

Supplementary References:

S1. Patil A, Salvatori R, Smith L, *et al.* Artificial intelligence-based reticulin proportionate area - a novel histological outcome predictor in hepatocellular carcinoma. *Histopathology* 2023; **83:** 512-525.

S2. Ricaurte Archila L, Smith L, Sihvo HK, *et al.* Performance of an Artificial Intelligence Model for Recognition and Quantitation of Histologic Features of Eosinophilic Esophagitis on Biopsy Samples. *Mod Pathol* 2023; **36:** 100285.

S3. Sivasubramaniam P, Stokes N, Patil A, *et al.* Digital Hepatic Iron Content: An Artificial Intelligence Model for Spatially Resolved Histologic Iron Quantitative Analysis in Liver Samples. *Lab Invest* 2023; **103:** 100200.

S4. Denic A, Alexander MP, Kaushik V, *et al.* Detection and Clinical Patterns of Nephron Hypertrophy and Nephrosclerosis Among Apparently Healthy Adults. *Am J Kidney Dis* 2016; **68:** 58-67.

S5. Denic A, Lieske JC, Chakkera HA, *et al.* The Substantial Loss of Nephrons in Healthy Human Kidneys with Aging. *J Am Soc Nephrol* 2017; **28:** 313-320.

S6. Denic A, Mathew J, Lerman LO, *et al.* Single-Nephron Glomerular Filtration Rate in Healthy Adults. *N Engl J Med* 2017; **376:** 2349-2357.

S7. Weibel ER, Gomez DM. A principle for counting tissue structures on random sections. *J Appl Physiol* 1962; **17:** 343-348.

S8. Maleszewski JJ, Lai CK, Veinot JP. Chapter 1 - Anatomic Considerations and Examination of Cardiovascular Specimens (Excluding Devices). In: Buja LM, Butany J (eds). *Cardiovascular Pathology (Fourth Edition)*. Academic Press: San Diego, 2016, pp 1-56.
